# Clinical management of alemtuzumab-induced autoimmune thyroid diseases: a narrative review

**DOI:** 10.1530/ETJ-25-0007

**Published:** 2025-06-10

**Authors:** Jacopo Manso, Ilaria Muller, Caterina Mian

**Affiliations:** ^1^ Endocrinology Unit, University Hospital S. Maria della Misericordia of Udine, Oncology Area Deparment, Azienda Sanitaria Universitaria Friuli Centrale (ASUFC), Udine, Italy; ^2^ Department of Clinical Sciences and Community Health, University of Milan, Milan, Italy; ^3^ Graves' Orbitopathy Center, Endocrinology Unit, Fondazione IRCCS Ca' Granda Ospedale Maggiore Policlinico, Milan, Italy; ^4^ Endocrinology Unit, Department of Medicine (DIMED), University of Padua, Padua, Italy; ^5^ Endocrine Unit, University Hospital of Padua, Padua, Italy

**Keywords:** thyroid, alemtuzumab, thyroid eye disease, graves’ disease, alemtuzumab-induced autoimmune thyroid diseases

## Abstract

Alemtuzumab is a powerful anti-CD52 drug that is an established treatment option in patients with multiple sclerosis due to its proven efficacy. However, in about 50% of patients, the use of alemtuzumab is burdened by the development of secondary autoimmune thyroid diseases, constituting a range of alemtuzumab-induced autoimmune thyroid diseases (AIATDs). Graves' disease (GD) is the most common AIATD, with an incidence of approximately 60%, and presents different characteristics from the conventional form. Indeed, GD with a fluctuating course is significantly more prevalent (15–50%), which poses a major challenge for physicians in its management. Other AIATDs also exhibit distinct features compared to their conventional counterparts; notably, hypothyroidism is frequently associated with TSH-receptor blocking antibodies, and alemtuzumab-induced GD demonstrates a higher rate of fluctuating course and potential for spontaneous remission. Alemtuzumab-induced thyroid eye disease (TED) is less common than conventional TED, with similar clinical and management characteristics. In this review, we summarize the latest evidence, also from real-world studies, with a focus on clinical management and possible predictors of AIATDs.

## Introduction

Multiple sclerosis (MS) is a debilitating chronic immune-mediated disease of the central nervous system (1).

Alemtuzumab is a powerful anti-CD52 monoclonal antibody that was formerly approved for the treatment of chronic lymphocytic leukemia, but it has been shown to be effective in treating MS, reducing relapses and disability, particularly in relapsing-remitting multiple sclerosis (RRMS) (2). Moreover, alemtuzumab has also been demonstrated to reduce relapses in patients with early active RRMS who have not received treatment previously (3). CD52 is a glycoprotein of unknown function found on T‐lymphocytes, B‐lymphocytes, and NK cells. Therefore, alemtuzumab induces a rapid and severe depletion of lymphocytes, followed by a differential recovery of lymphocyte subsets, with long-lasting suppression of CD4/CD8+ T-cells compared with B‐cells, making it an efficient treatment for T-cell-driven diseases (4, 5). The standard treatment consists of a first cycle of 12 mg/day iv for 5 consecutive days, followed (usually a year later) by a second cycle of 12 mg/day iv for 3 consecutive days (with optional supplementary cycles based on clinical or radiological evidence of disease), without additional long-term immunosuppressive drugs needed (6). Alemtuzumab has been authorized since 2013 for the treatment of RRMS in many countries, including the United States and the European Union, due to its proven efficacy. The main adverse effect of alemtuzumab is the development of secondary autoimmune diseases, including hematological (e.g. immune thrombocytopenic purpura), renal (e.g., anti-glomerular basement membrane disease), and dermatological conditions (7). However, the development of alemtuzumab‐induced autoimmune thyroid diseases (AIATDs) is the most frequent adverse effect following the administration of this treatment.

Of these secondary autoimmune phenomena, thyroid dysfunction is consistently reported as the most frequent, affecting a significant proportion of treated individuals (7).

This narrative review aims to summarize the current understanding of the pathophysiology, clinical presentation, and management of AIATDs, incorporating evidence from clinical trials and real-world studies, with a focus on aspects relevant to clinical practice.

## Literature search strategy

This narrative review is based on the literature search conducted on PubMed/MEDLINE for articles published up to December 2024. Search terms included ‘alemtuzumab’, ‘thyroid’, ‘Graves' disease’, ‘thyroiditis’, ‘hypothyroidism’, ‘thyrotoxicosis’, ‘thyroid eye disease’, ‘Graves' orbitopathy’, ‘alemtuzumab-induced autoimmune thyroid diseases’ and ‘alemtuzumab-induced autoimmune diseases’. Relevant clinical trials, real-world studies, meta-analyses, case series, and guidelines published in English were selected based on their relevance to the clinical management and pathophysiology of AIATDs. Priority was given to peer-reviewed publications indexed in PubMed.

## Alemtuzumab-induced autoimmune thyroid diseases

### Pathophysiology

AIATDs are the most common adverse effect observed in patients diagnosed with RRMS. AIATDs are generally considered part of the ‘immune reconstitution syndrome’, occurring after the recovery of immune cell numbers following a drug-induced depletion phase (8). Interestingly, AIATDs have been described less frequently in patients treated with alemtuzumab for rheumatoid arthritis or leukemia, despite different dosing regimens used in those conditions, suggesting a specific role of the MS autoimmune background in their development (9, 10, 11).

**Figure 1:**
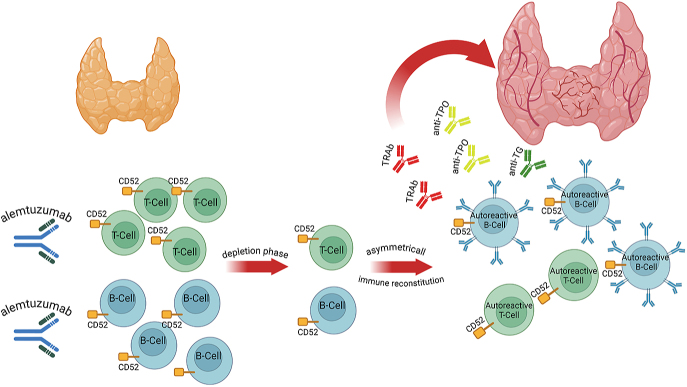
Pathophysiology of alemtuzumab‐induced autoimmune thyroid diseases. Anti‐TG, anti‐thyroglobulin; anti‐TPO, anti-thyroperoxidase; TRAb, TSH‐receptor antibodies.

The underlying pathophysiology for these events remains to be fully elucidated; however, the prevailing hypothesis is rooted in the specific pharmacodynamics of alemtuzumab. In fact, alemtuzumab causes a depletion of lymph cells, with a delay in the repopulation of T-cells compared with B-cells, with a possible proliferation of abnormal autoreactive B- and T-cells due to an overproduction of IL-21, potentially leading to a broader spectrum of autoantibody production, including both stimulating and blocking TRAb Fig. 1 (12, 13). The differential kinetics of T-regulatory and effector cell recovery likely contribute to this loss of self-tolerance (13).

### Epidemiology and clinical course

In the pivotal phase 3 (CARE-MS I and II) and phase 2 (CAMMS223) randomized controlled trials, which compared alemtuzumab to interferon beta-1a in patients with RRMS and established its efficacy, the overall incidence of AIATDs was approximately 36% (14, 15). In 2019, a meta-analysis on this topic, which included a total of 1,362 MS patients treated with alemtuzumab from seven studies, revealed that 33% of these patients experienced AIATDs during follow-up (16).

In general, the large majority of AIATDs were observed within the first 3 years following the initial alemtuzumab infusion, with the incidence reaching its peak at year 3, or 16–23 months after the last alemtuzumab dose (mainly in the first 5 years after the last infusion) (6, 8, 14).

Graves' disease (GD) is the most common AIATD, responsible for approximately 60% of all documented cases (16). More frequently than conventional forms, between 15 and 50% of cases showed a fluctuating course (F-GD), defined as GD with unexpected fluctuations between hyper- and hypothyroid states (or vice versa), not attributable to overtreatment or poor treatment compliance, but due to the presence of TSH-receptor antibodies (TRAb) with both stimulatory and inhibitory effects (16, 17). The second most common AIATD (about 15%) is primary hypothyroidism due to the development of Hashimoto’s thyroiditis or to the presence of inhibitory TRAb that block TSH receptor signaling (blocking TRAb) (16). In contrast to conventional hypothyroidism, where they are present in only 10% of cases, TRAb are detected in 30–50% of patients with alemtuzumab-induced hypothyroidism, and the subpopulation of blocking TRAb is the pathophysiological mechanism responsible (6, 14, 18). In some cases, TRAb-positive hypothyroidism might be the first sign of F-GD. The final and less common AIATD is a silent thyroiditis form (characterized by transient thyrotoxicosis due to thyroid destruction, followed by hypothyroidism or recovery, typically with low radioiodine uptake), which accounts for approximately 10% of cases (16). In 30% of cases, a patient may experience different episodes of thyroid dysfunction during follow-up (14).

Recent studies with extended follow-up periods or real-life cohorts have revealed that AIATDs occur with even greater frequency, ranging from 39 to 55%, and with earlier onset, ranging from 17 to 26 months after the first course of alemtuzumab and 10 to 12 months after the last (17, 19, 20, 21, 22).

Interestingly, in real-world series, the prevalence of F-GD seems to be higher (41–78%) than in trials (15%) (17, 20, 21, 22).

Considering the higher prevalence of F-GD and TRAb-positive hypothyroidism, AIATDs exhibit distinctive clinical and immunological features that differ from those observed in conventional cases affecting the general population, thus rendering the clinical management of the disease a challenging endeavor. Moreover, unlike conventional thyroid disease, between 12 and 20% of alemtuzumab-induced GD had spontaneous remission, and 14% of Hashimoto’s thyroiditis showed a transition to hyperthyroidism (vs 1.2% reported in the general population) (14, 16, 19).

A controversial issue in the existing literature concerns the discrepancy in the remission rate of AIATDs reported in trials and real-world studies. In fact, Dayan *et al.* in their re-evaluation of patients in the phase 3 CARE-MS I, II, and extension studies over 6 years, showed that the remission rate of AIATDs was better than it seemed and was around 54.8%, more similar to conventional GD (19). A remission rate of 40% was also observed in the large Norwegian 2024 retrospective study (median follow-up time of 72 months) by Ueland *et al.*, with a median thyroid treatment time of 22 months (22).

Conversely, in the real-life studies by Manso *et al.*, Kazakou *et al.*, and Hansen *et al.*, the remission rate of AIATDs was lower (18, 11, and 22%), with a mean follow-up period of 32, 43.5, and 43 months, respectively (17, 20, 21). Some potential explanations for this discrepancy may be the shorter follow-up period of the latter real-world studies or the higher frequency of the F-GD phenotype.

The question of whether alemtuzumab-induced GD is more indolent or more aggressive than conventional GD remains debated.

Compared to conventional GD, alemtuzumab-induced GD is characterized by several distinct features impacting management: a significantly higher prevalence of a fluctuating course (F-GD), attributed to the potential co-existence or temporal shift between stimulating and blocking TRAb (6, 16, 17, 18); a possibly different remission rate profile, with some studies suggesting lower long-term remission off antithyroid drugs compared to conventional GD, particularly in real-world cohorts (17, 20, 21), although longer-term trial data suggest rates may become comparable over many years (19); and a frequent occurrence of TRAb-positive hypothyroidism, which may precede or follow a hyperthyroid phase (6, 14, 18). These differences underscore the unique challenges in managing AIATD-related GD.

Some reports on case series have suggested a more favorable outcome (7, 14, 19, 22), while others have indicated a more challenging prognosis due to the higher fluctuating and unpredictable course of the disease, and a low remission rate compared to conventional forms (6, 17, 20, 21).

From a neurological point of view, the development of AIATDs was not a predictor of the efficacy of alemtuzumab for RRMS in clinical trials (3, 19). However, Ueland *et al.* observed in their recent real-world study a lower frequency of new MS relapses and radiological lesions in alemtuzumab-induced GD patients compared to those without (22).

Specific data on whether optional additional cycles of alemtuzumab impact the clinical course or relapse rate of pre-existing AIATDs are limited, although continued monitoring remains essential (19). Finally, given the limited data in the literature, it is not possible to determine a possible relationship between thyroid cancer and treatment with alemtuzumab.

### Clinical management

According to the most recent European Thyroid Association (ETA) guidelines, patients receiving alemtuzumab should undergo blood thyroid testing at 3-month intervals for a period of 4 years after the last alemtuzumab dose (8).

Thyrotoxicosis is the most prevalent AIATD, yet it is imperative to elucidate the etiology, distinguishing GD from destructive forms, to establish the most appropriate treatment. In the case of a patient with alemtuzumab-induced thyrotoxicosis, first the TRAb assay should be requested in addition to anti-thyroperoxidase (anti-TPO) and anti‐thyroglobulin (anti-TG) antibodies. Thyroid ultrasonography is also a valuable diagnostic tool in this context. In case of a positive TRAb test, a diagnosis of GD can be made (6, 14). Conversely, if the TRAb are negative, thyroid scintigraphy should be performed to differentiate between the two forms, with GD showing a diffuse tracer uptake, while destructive thyroiditis shows a reduced uptake (23). In the case of alemtuzumab-induced primary hypothyroidism, complete thyroid autoantibodies (TRAb, anti-TPO, and anti-TG) should be assessed. Indeed, the presence of TRAb in hypothyroid patients has been shown to identify those at risk of developing F-GD, thus highlighting the necessity for a more intensive biochemical surveillance strategy. Moreover, TRAb positivity in hypothyroid patients is clinically important, as hypothyroidism due to blocking TRAb, rather than destruction of the thyroid gland, may be potentially reversible.

The ETA guidelines suggest monthly monitoring of thyroid function for both thyrotoxicosis and hypothyroidism before the commencement of any treatment, given that approximately 20% of AIATDs are only transient or show a fluctuating course, unless the patient is symptomatic or at high risk (pregnancy, cardiovascular disease), or if thyroid dysfunction persists >3 months (8).

Most patients with alemtuzumab-induced GD responded to antithyroid drugs (6, 14, 17). Out of 450 patients in a meta-analysis, 56% required only antithyroid drugs, 22% needed additional RAI, and 11% underwent definitive surgery (16).

In the context of GD, the fluctuating phenotype is particularly challenging to treat due to the difficulty of reaching a stable euthyroid state with medications. Unexpected fluctuations between hyper- and hypothyroid states, not attributable to overtreatment or poor treatment compliance, are triggered by the coexistence of stimulatory and blocking TRAb, as demonstrated in some reports (6, 18). The 2019 guideline and the most recent real-life studies both suggest that block and replace (antithyroid drugs plus levothyroxine) therapy is the most effective option in cases of F-GD (8, 17, 22).

Because of the uncertainty surrounding the response to treatment, it is reasonable to use definitive treatments (surgery or radioiodine treatment) for alemtuzumab-induced GD in circumstances such as those of conventional GD. Patients with overt or clinical hypothyroidism should start treatment with levothyroxine according to the general guidelines. In alemtuzumab-induced silent thyroiditis, a conservative approach with only symptomatic therapy (beta blockade) is recommended.

Finally, given the limited data in the literature, it is not possible to determine a possible relationship between thyroid cancer and treatment with alemtuzumab.

### Management considerations in pregnancy

Given that RRMS predominantly affects women of childbearing age, the management of AIATDs during conception, pregnancy, and the postpartum period warrants careful consideration. The ETA guidelines suggest that women pregnant or seeking pregnancy within 4 years of the last alemtuzumab dose should have their thyroid function monitored more frequently, ideally monthly (8). Immediate referral to an endocrinologist is recommended as soon as AIATDs develop to ensure appropriate treatment and specialized monitoring throughout pregnancy. For women with alemtuzumab-induced hypothyroidism seeking pregnancy, levothyroxine should be continued to maintain TSH in the normal range as per general pregnancy guidelines, postponing any trial off levothyroxine until after delivery and breastfeeding.

Real-world data on pregnancy outcomes in patients with AIATDs are limited but emerging. A Norwegian cohort study reported four successful pregnancies in patients with active alemtuzumab-induced GD, with all patients remaining euthyroid and no maternal or fetal complications noted. Three of these women had TRAb levels more than three times the upper reference limit throughout pregnancy. Two received levothyroxine (presumably for an F-GD course), and one received propylthiouracil in the first trimester, while the fourth, treated with propylthiouracil until pregnancy, normalized TRAb and required no thyroid medication during gestation (22). A Greek prospective study detailed two successful pregnancies in alemtuzumab-treated patients (20). One patient developed Hashimoto’s thyroiditis with hypothyroidism 11 months post-first alemtuzumab cycle; her levothyroxine dose was slightly increased during pregnancy, and she delivered a healthy child. The second patient developed GD with high TRAb levels during the fifth week of pregnancy (32 months post-first alemtuzumab). She was treated with propylthiouracil, which was stopped at the end of the first trimester as hyperthyroidism resolved and TRAb declined. A relapse of mild hyperthyroidism at gestational week 18 necessitated low-dose carbimazole, which was subsequently discontinued at week 25 as TRAb further declined. The pregnancy resulted in a healthy, euthyroid neonate. This case highlights that alemtuzumab-induced GD can develop during pregnancy and may, in some instances, ameliorate, allowing antithyroid drug discontinuation, similar to conventional GD. However, other reports, including those referenced in a Danish cohort study, describe cases of alemtuzumab-induced GD worsening or having a more aggressive course during pregnancy, with high antithyroid drug requirements and potential neonatal thyrotoxicosis, particularly if GD onset is during pregnancy (21).

Given the potential for AIATDs, particularly GD, to arise or fluctuate during pregnancy, pre-conception counseling and close multidisciplinary management involving neurologists, endocrinologists, and obstetricians are crucial for optimizing maternal and fetal outcomes. Monitoring of TRAb levels is important due to their potential to cross the placenta and cause fetal or neonatal thyroid dysfunction, especially if maternal TRAb levels are significantly elevated (20).

## Risk factors for alemtuzumab-induced autoimmune thyroid diseases

Identifying from the outset those MS patients at higher risk of developing AIATDs would be of great clinical benefit in customizing closer follow-up in order to intercept early the development of thyroid problems. A number of studies have tried to isolate possible predictive factors, and the results are summarized in Table 1.

**Table 1:** Summary of risk factors for alemtuzumab‐induced autoimmune thyroid events.

Study	Year	Patients, *n*	Follow-up, months^§^	Risk factors for AIATDs	Independent risk factor for AIATDs
Manso *et al.* (17)	2021	57	32 ± 17	• Baseline anti-TG and anti-TPO positivity • Baseline higher absolute levels of anti-TG and anti-TPO	• Baseline anti-TPO antibody positivity
Daniels *et al.* (14)	2014	216	57 (max: 81)	• Female gender • Baseline weight below 71 kg • Age <31 years	
Cossburn *et al.* (7)	2011	248	34 (7–107)	• Family history of autoimmune disease^†^ • Smoking history^‡^	• Family history of autoimmune disease • Smoking history
Azzopardi *et al.* (26)	2015	141	Not known	• Serum interleukin-21	
Yap *et al.* (25)	2020	52	55 ± 29	• MS brainstem involvement at diagnosis* • Age <32 years*	
Muller *et al.* (18)	2018	45	108 ± 30	• Baseline anti-TPO positivity • Custom-made TRAb testing (not automated) positivity	
Ruck *et al.* (24)	2019	106	36 (ad interim analysis)	• Baseline anti-TG and anti-TPO positivity	• Baseline anti-TPO and anti-TG positivity
Ueland *et al.* (22)	2024	183	72 (48–102)	• Female gender	

AIATDs, alemtuzumab‐induced autoimmune thyroid events; anti‐TG, anti‐thyroglobulin; anti‐TPO, anti‐thyroperoxidase.

* Risk factor for symptomatic GD vs other AIATDs.

^†^ Data on 231 patients.

^‡^ Data on 196 patients.

^§^ Values are mean ± SD or median (IQR).

In 2011, Cossburn and co-workers conducted a large prospective analysis of patients from multiple UK centers not included in CAMMS clinical trials (7). This study identified smoking habits and family history of generic autoimmune diseases as potential risk factors for the development of AIATDs, respectively with an odds ratio (OR) of 3.05 and 7.31. While gender and age (well-known risk factors for autoimmune thyroid diseases) were not found to represent possible threat elements in this cohort. In contrast, the 2014 study by Daniels *et al.* involved a single endocrinologist who reassessed thyroid events in patients followed for a median of 80 months in the CAMSS23 trial. They found that the female gender (OR 2.37), a baseline weight below 71 kg (OR 2.33), and an age below 31 years (OR 1.88) were all significant predictors of developing AIATDs (14). In the recent Norwegian retrospective study of 183 MS patients treated with alemtuzumab, Ueland *et al.* confirmed that female sex was identified as a risk factor for AIATDs with an OR of 3.9 (22). However, other studies found no association with gender, age, smoking status, or family history of thyroid disease (17, 20, 24).

From a different perspective and with the limitations of a retrospective study in a small population of MS patients, Yap *et al*. found that 83% of symptomatic alemtuzumab-induced GD patients presented clinical and radiological evidence of brainstem involvement at diagnosis (25). Therefore, brainstem involvement conferred an 11-fold higher risk of developing alemtuzumab-induced symptomatic GD.

Pretreatment positivity for anti-thyroid antibodies, particularly anti-TPO, has been demonstrated to be a significant predictor of the development of AIATDs. In a prospective study of 106 MS patients treated with alemtuzumab, Ruck *et al.* found that anti-TPO and anti-TG positivity at baseline were associated with a higher risk of AIATDs and also a shorter time to onset of AIATDs (24). In fact, 48% of patients who developed AIATDs during follow-up presented anti-TPO, anti-TG, or a combination of these at baseline, whereas only 5% of patients without AIATDs presented a positive pretreatment anti-thyroid antibody titer. The independent predictive significance of anti-TPO and anti-TG was also confirmed in multivariate regression analysis. In our retrospective monocentric study conducted in 2021, pretreatment anti-TG and anti-TPO positivity, as well as baseline higher absolute titers of anti-TG and anti-TPO, emerged as risk factors for developing AIATDs, but TRAb positivity did not (17). Indeed, AIATDs developed in 59% of patients with pretreatment anti-TG or anti-TPO positivity, while these events occurred in only 12% of patients with negative anti-thyroid antibodies. However, the absolute titer of TRAb has shown a potentially interesting clinical utility. Higher levels of TRAb at the time of GD diagnosis have been shown to correlate strongly with an increased risk of the F-GD form. Furthermore, in the same work, we revealed through ROC curve analysis that a threshold of 7.3 IU/L could be useful in predicting the risk of developing F-GD, with a positive predictive value of 100%. Thus, TRAb levels measured with commercially available automated assays at the diagnosis of alemtuzumab-induced GD may be a novel biomarker for predicting a fluctuating disease phenotype. However, we need to be aware of the limitations of this TRAb cut-off, which was derived from a small series of eight patients (17). Additional validation is needed for reliable use in routine clinical practice.

In a further longitudinal retrospective analysis of a cohort of 45 alemtuzumab-treated patients in the United Kingdom, baseline anti-TPO positivity in combination with the detection of TRAb with custom-made assays was found to be able to predict the development of AIATDs (20% of hyperthyroid cases and 80% of hypothyroid cases), while using commercially available automated TRAb measurements did not have this capability (18). Thus, AIATDs developed in 100% of baseline custom‐made TRAb positive and/or anti-TPO positive patients, as opposed to 39.1% baseline custom‐made TRAb negative and/or anti-TPO negative. However, an in-house assay for TRAb is not easily available in daily clinical practice.

It is important to note, however, that the ETA guidelines stated that routine measurement of anti-TPO or TRAb before alemtuzumab therapy is not suggested, despite the increased risk of AIATDs in positive patients (8). This seems reasonable because, for example, in the study by Daniels *et al.*, 85% of patients who developed AIATDs were anti-TPO negative at baseline (14).

Finally, an intriguing study published in 2015 by Azzopardi *et al.* identified serum interleukin-21 (IL-21) measured at baseline as a predictor of the development of AIATDs in MS patients, with a sensitivity of 81% and specificity of 67%, using a threshold concentration of 230 pg/mL (26). However, IL-21 has been shown to be a valid biomarker for the development of AIATDs only when measured using an enzyme-linked immunosorbent assay (ELISA) kit (3A3-N2, detection antibody 2B2-G20 made by ascites), which, however, has been discontinued since 2012, but not with currently commercially available ELISA methods.

## Alemtuzumab-induced thyroid eye disease

Thyroid eye disease (TED), also known as Graves’ orbitopathy, is an orbital autoimmune complication which affects around 30–50% of patients with conventional GD (27, 28). Orbital cells, including fibroblasts, express thyroid-stimulating hormone receptor and overexpress the insulin-like growth factor-1 receptor (IGF-1R); both molecules seem crucial in TED pathogenesis, even if the immune mechanisms are still far from being fully elucidated (29). TED is characterized by complex orbital processes including immune infiltration, adipose tissue expansion, and fibroblast proliferation with production of glycosaminoglycans, leading to orbital inflammation, edema, proptosis and extraocular muscle enlargement and impairment with consequent diplopia (30). Conventional TED is usually mild, with moderate-to-severe forms affecting around 5–6% of GD patients. Sight-threatening forms are rare, including dysthyroid optic neuropathy caused by optic nerve compression, and corneal ulceration or globe prolapse due to severe exophthalmos (31, 32, 33).

The incidence of alemtuzumab-induced TED (AITED) has been described in only 13–16% patients with alemtuzumab-induced GD, appearing lower than the conventional form (6, 34, 35). Published case series were retrospective and patients were not routinely assessed by dedicated ophthalmologists, thus the real incidence of AITED might be underestimated. Indeed, the observed AITED forms were around 50% mild and 50% moderate-to-severe, thus reflecting the tendency to skip ophthalmology referral for paucisymptomatic cases (6, 34, 35, 36). As further confirmation, a small prospective study of patients treated with alemtuzumab who systematically underwent ophthalmological evaluation detected an incidence of AITED in 4/9 (44.4%) patients with alemtuzumab-induced GD (20).

To our knowledge, so far only 44 cases of AITED have been reported as small case series, and no significant clinical and management differences with the conventional forms of TED were highlighted (22, 34, 35, 36). The clinical course of TED is usually characterized by an early active phase of florid inflammation, followed by a chronic phase of fibrosis (33). Mild TED cases are usually addressed with topical and lifestyle-modifying measures, and the use of selenium for active forms.

Management of moderate-to-severe active TED typically involves systemic immunotherapy, primarily intravenous glucocorticoids (ivGC) as first-line therapy, followed by rehabilitative surgery in the chronic phase (31). The increasing use of novel targeted therapies for TED (e.g., rituximab, tocilizumab, teprotumumab) requires particular attention in patients already treated with a panlymphopenic agent such as alemtuzumab to avoid potential pharmacological interference and toxicity. So far, no detailed studies have been conducted to evaluate the long-term efficacy and safety of the use of sequential biological drugs in patients with AITED. Small case series have reported the successful use of anti-IL6 agents (tocilizumab or sarilumab) and teprotumumab in, respectively, four and one patients with AITED, leading to disease inactivation and proptosis improvement; however, details about long-term safety are missing (35, 36). Regarding oral immunosuppressants, Roos *et al.* reported the combined use of steroids and cyclosporine in three patients with severe AITED, which was successful in two cases, while the third patient was later switched to tacrolimus and mycophenolate due to poor response (34). Recently, Muller *et al.* described a case of active moderate-to-severe AITED developed 3 years after alemtuzumab therapy for MS. After an unsuccessful course of ivGC, the patient was effectively treated with rituximab as second line therapy, followed by disease inactivation. Interestingly, the authors also provided details about circulating and tissue-resident immunophenotyping at several time points before and after treatment. At the time of rituximab therapy (4 years following alemtuzumab), the patient’s B-cell count had not fully recovered from alemtuzumab-induced lymphopenia; however, the treatment was successful and AITED was stably inactivated. Ten months after rituximab, despite the B-cell numbers being still fully depleted as a likely cumulative effect of consecutive alemtuzumab and rituximab treatments, the patient had not developed any infection, and both her MS and AITED remained in remission (37). These data are encouraging in terms of both efficacy and safety of consecutive immunotherapies for AITED; however, dedicated studies with higher numbers of patients need to be conducted. In particular, it would be valuable to monitor lymphocyte counts and other inflammatory markers, as well as long-term rates of AITED and MS relapses or reactivations, and incidence of side effects such as infections or neoplastic processes.

## Conclusion

AIATDs are a very common complication in patients with RRMS treated with alemtuzumab, with a peak occurrence between 2 and 3 years after the initial dose. GD remains the most frequently observed AIATD following alemtuzumab treatment. AIATDs show peculiar clinical aspects compared with conventional thyroid autoimmune diseases in the general population. In particular, the F-GD phenotype is very common and represents a clinical challenge for which block and replace treatment protocol is often recommended. The incidence of AITED appears to be lower than that of the conventional form. Management of AIATDs requires careful monitoring and individualized strategies, particularly considering the potential for a fluctuating course and the specific context of MS treatment. Special considerations are needed for managing AIATDs during pregnancy. To date, AITED should be treated in accordance with the latest general disease-specific guidelines. Patients with pretreatment presence of anti-thyroid antibodies, particularly anti-TPO, are at increased risk of developing AIATDs. Whether alemtuzumab-induced GD has a more favorable or worse prognosis than conventional GD remains an open question, influenced by factors such as fluctuating course and treatment response. Long-term antithyroid drugs may be required, and definitive treatments (radioiodine or surgery) should be pursued in circumstances similar to those of conventional GD.

## Declaration of interest

The authors declare that there is no conflict of interest that could be perceived as prejudicing the impartiality of the research reported.

## Funding

This research did not receive any specific grant from any funding agency in the public, commercial, or not-for-profit sector.

## Author contribution statement

JM contributed to the conception and design of the study. JM wrote the first draft of the manuscript. JM, IM, and CM wrote sections of the manuscript. All authors contributed to revising the manuscript, and read and approved the submitted version.
